# Endoscopic-Assisted Tonsillectomy With Preemptive Hemostasis and Low-Output Bipolar Energy: A Strategy to Reduce Postoperative Hemorrhage

**DOI:** 10.7759/cureus.84933

**Published:** 2025-05-27

**Authors:** Haruka Ota, Takuya Yoshida, Keita Suzuki, Kazutake Yagi, Kenjiro Higashi, Akira Ohkoshi, Yukio Katori

**Affiliations:** 1 Department of Otolaryngology, Iwate Prefectural Iwai Hospital, Ichinoseki, JPN; 2 Department of Otolaryngology-Head and Neck Surgery, Tohoku University Graduate School of Medicine, Sendai, JPN

**Keywords:** endoscopic, postoperative hemorrhage, preemptive hemostasis, surgical procedure, tonsillectomy

## Abstract

Introduction

Tonsillectomy is a common surgical procedure in otolaryngology; however, postoperative hemorrhage remains a serious complication. Although novel hemostatic devices have been introduced, no definitive preventive strategy has been established. This study evaluated a standardized surgical approach combining preemptive hemostasis, reduced energy device output, and endoscopic assistance to minimize postoperative complications.

Methods

This single-center, non-randomized, observational cohort study included 137 patients aged 15 years or older who underwent extracapsular tonsillectomy between April 2018 and March 2025. Patients were grouped based on the time period of treatment: those who underwent conventional tonsillectomy between 2018 and 2021 comprised the Conventional Group (n = 85), and those who underwent a standardized procedure incorporating preemptive hemostasis and low-output energy devices between 2022 and 2025 comprised the Standardized Group (n = 52). Perioperative outcomes, postoperative hemorrhage rates, pain control, and hospital stay length were compared between groups using the Mann-Whitney U test and Fisher’s exact test (p < 0.05 was considered significant).

Results

The Standardized Group showed a significantly lower postoperative hemorrhage rate (0% vs. 9.5%, p = 0.0236) and less intraoperative blood loss (0.42 ± 1.01 mL vs. 8.31 ± 26.7 mL, p = 0.0031) compared to the Conventional Group. Although operative time was longer (83.5 ± 33.3 min vs. 56.5 ± 23.8 min, p < 0.001), the Standardized Group had a shorter hospital stay (8.86 ± 1.24 days vs. 10.1 ± 1.15 days, p < 0.001) and required fewer rescue analgesic interventions (p = 0.0178).

Conclusion

The combination of preemptive hemostasis, reduced energy output, and endoscopic supervision significantly improved surgical outcomes, reduced complications, and enhanced the educational experience for junior surgeons. This protocol represents a standardized, cost-effective technique that enhances safety and surgical training.

## Introduction

Tonsillectomy is one of the most frequently performed procedures in otolaryngology, primarily conducted for the treatment of chronic tonsillitis and peritonsillar abscess [1]. Recently, interest in tonsillectomy has increased due to the rising incidence of human papillomavirus (HPV)-related oropharyngeal cancer [2], with evidence suggesting a potential role in cancer prevention [3]. Additionally, complete removal of the palatine tonsils is indicated for the treatment of IgA nephropathy and palmoplantar pustulosis [4-6]. Diagnostic tonsillectomy also plays a crucial role in identifying unknown primary cancer (UPC) of the head and neck [7]. Therefore, establishing a reliable extracapsular tonsillectomy technique is essential. Postoperative bleeding and pain remain major challenges, with bleeding potentially leading to fatal outcomes, thereby emphasizing the need for effective hemostasis [1,8,9]. Energy devices are commonly used for intraoperative hemostasis [10]; however, no consensus has been established regarding the optimal energy output, and decisions are often left to the surgeon’s discretion, risking excessive tissue damage. Conversely, meticulous preemptive hemostasis and the use of low-output energy devices may reduce tissue damage and postoperative complications [11].

In many countries, including Japan, tonsillectomy is a procedure commonly experienced by young otolaryngology residents during the early stages of their surgical training [12,13]. However, due to the anatomical characteristics of the palatine tonsils, the surgical field is confined within the oral cavity, which is narrow and challenging for shared visualization. Furthermore, consensus regarding complication reduction has not yet been established, and variations in techniques and instruments among institutions hinder standardization. These circumstances and limitations are among the factors contributing to the difficulty in establishing a safe and standardized surgical technique. 

A notable contribution to the refinement of tonsillectomy techniques was made by Andrea, who introduced the use of bipolar cautery under microscopic guidance to achieve precise hemostasis with minimal thermal injury [14]. His approach demonstrated the benefits of combining surgical microscopy and controlled coagulation, resulting in reduced intraoperative bleeding and postoperative complications. While his method emphasized visual clarity and tissue preservation, it has not been widely adopted or standardized in contemporary practice. This study compares conventional techniques with the use of low-output energy devices combined with preemptive hemostasis, evaluating postoperative bleeding, pain, and surgical invasiveness. In addition, we examined the educational impact on young surgeons and the safety benefits associated with endoscopic supervision by senior surgeons. In a search of the literature from 2000 to 2025 using the keywords “tonsillectomy” and “postoperative bleeding,” no studies could be identified that investigated the impact of energy device output limitations or the use of endoscopic assistance on postoperative bleeding. This indicates that our study provides a novel perspective and has the potential to offer new insights into reducing the risk of postoperative bleeding, as well as improving postoperative pain.

## Materials and methods

Study design

This single-center, non-randomized observational cohort study included adult patients who underwent tonsillectomy between 2018 and 2025 at Iwate Prefectural Iwai Hospital, Ichinoseki, Japan. The study protocol was approved by the Iwate Prefectural Iwai Hospital Ethics Committee (approval number: R6-48). The study was conducted in accordance with the Declaration of Helsinki.

Study population

Japanese patients aged 15 years or older, diagnosed with either chronic tonsillitis or IgA nephropathy, who underwent tonsillectomy under general anesthesia at the Department of Otorhinolaryngology, Iwate Prefectural Iwai Hospital, between April 2018 and March 2025, were enrolled in this observational cohort study. 

Patients treated between April 2018 and December 2021 underwent conventional tonsillectomy and were retrospectively analyzed as a historical control group. In January 2022, a novel standardized surgical protocol was introduced, and patients treated between 2022 and 2025 under this protocol were prospectively observed with predefined surgical steps and outcome measures. Written informed consent was obtained from all patients in the prospective cohort.

Study groups

Patients were divided into two groups according to the time period of their treatment, which determined the surgical technique used.

Conventional Group

This group included patients who underwent tonsillectomy between April 2018 and September 2022 (n=85). In all cases, extracapsular tonsillectomy was performed using blunt dissection along the peritonsillar plane, followed by bipolar cauterization of bleeding points after tonsil removal. The output settings of the energy device were determined at the discretion of the operating surgeon, with no standardized measures to minimize tissue charring. A total of eight surgeons performed procedures in this group.

Standardized Group

This group included patients who underwent tonsillectomy between October 2022 and May 2025 (n=52). A standardized surgical protocol was strictly followed. Preemptive hemostasis was performed in all cases using low-output bipolar energy. In cases without significant adhesions, preemptive coagulation of key vessels and surrounding tissues was followed by gentle extracapsular blunt dissection along the plane closest to the tonsillar capsule. In contrast, when adhesions made blunt dissection difficult, sharp dissection with scissors was performed instead, again following preemptive hemostasis. Particular care was taken in all cases to preserve the pharyngeal constrictor muscle within the tonsillar fossa whenever possible.

Standardized surgical procedure

**Figure 1:**
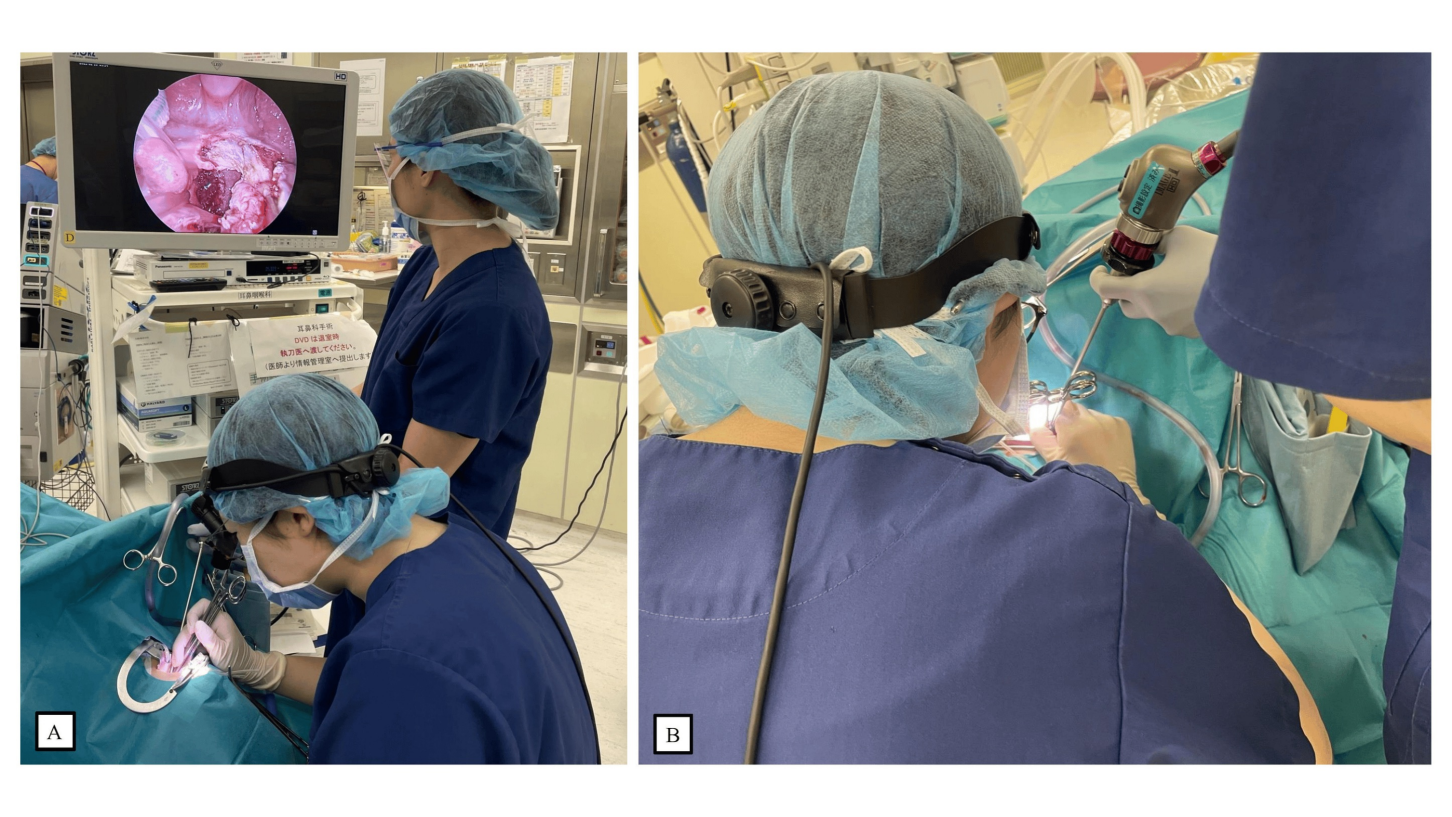
Endoscopic system and surgical setup (A) Setup showing the monitor, surgeon, and assistant positions; (B) Surgeon’s hand position relative to the endoscopic camera.

The output of the bipolar energy device was limited to a maximum of 12 W. If macroscopic signs of tissue carbonization or fluid boiling were observed, the output was further reduced in accordance with the protocol. A senior surgeon provided real-time supervision and feedback via endoscopic observation. Although endoscopic monitoring was utilized for supervisory purposes, the primary surgeon completed all surgical steps under direct visualization, without reliance on the endoscopic monitor. The endoscope was inserted from the oral commissure contralateral to the tonsil being dissected, which helped prevent physical interference with the surgeon’s hands or tools. Additionally, the camera was aligned as parallel as possible to the direction of the traction forceps (Pean clamp), allowing for a clear view of the dissection plane without obstructing the operative field. Figure 1A illustrates the positions of the monitor, the primary surgeon, and the assistant during the procedure. Figure 1B demonstrates the spatial relationship between the surgeon’s hands and the endoscopic camera system. Essential parts of the procedure are shown in Videos 1-3. All surgeries in this group were performed by three surgeons trained in the standardized protocol. The surgeons involved in each group were entirely distinct, with no overlap between the two groups. The standardized surgical protocol is given in Table 1.

**Video 1:** Mucosal incision and identification of the tonsillar capsule A sharp incision was made on the tonsillar side, medial to the anterior palatal arch. Hemostasis was achieved using bipolar cautery while gently exposing the tonsillar capsule at the incision site.

**Video 2:** Tonsillar capsule dissection without adhesions The tonsil is gently retracted using bipolar forceps, preserving the pharyngeal constrictor fascia within the tonsillar fossa.

**Video 3:** Management of fibrous adhesions due to prior inflammation Adhesive tissue is cauterized and dissected as close to the tonsillar side as possible to avoid excessive tissue removal and preserve the capsule plane.

**Table 1:** Surgical procedure description

Step	Surgical Procedure Description	Corresponding Media
1	The anterior and posterior palatal arches were retracted to assess the relationship between the mucosa, palatal arches, and palatine tonsils.	—
2	The tonsil was grasped with a Pean forceps and gently retracted to evaluate the degree of embedding. A submucosal injection of 0.5% lidocaine hydrochloride with epinephrine (1:100,000) was administered to create a bulge. A sharp incision was made on the tonsillar side of the anterior palatal arch.	Video 1
3	Hemostasis was achieved at the incision site using bipolar cautery while exposing the tonsillar capsule.	—
4	Following the exposed capsule, preemptive bipolar coagulation and sharp dissection with scissors were performed in the superior and inferior directions.	—
5	In cases without adhesions, preemptive hemostasis was performed using low-output bipolar energy on key vessels and surrounding tissues prior to dissection. Gentle traction and dissection using bipolar forceps then allowed for peeling along the tonsillar capsule, preserving the pharyngeal constrictor fascia within the tonsillar fossa. This process was repeated as needed.	Video 2
6	If adhesions due to prior inflammation were present, the fibrous tissue was cauterized and dissected as close to the tonsil side as possible. Dissecting away from the capsule makes it difficult to identify the correct plane and may result in excessive and unnecessary tissue removal.	Video 3
7	If excessive carbonization was observed during dissection, the bipolar output was reduced by 1 watt increments.	—
8	Steps 5 to 7 were repeated along the capsule to complete the tonsil removal. Mouth gag loosened every 30 minutes to reduce risk of sensory/taste disturbance.	—
9	Hemostasis was confirmed after removal. If preemptive coagulation was effective, additional cauterization at this stage was generally unnecessary.	—

Surgical instruments

In both groups, a bipolar energy device combined with bipolar forceps was used. Specifically, either the VIO® 3 system (Erbe Elektromedizin GmbH, Tübingen, Germany) in softCOAG® mode with Premium Bipolar Forceps (Erbe Elektromedizin GmbH) or the CONMED System 5000™ Electrosurgical Generator (CONMED Corporation, Largo, Florida, United States) with SuperGliss® non-stick Bipolar Forceps (Sutter Medizintechnik GmbH, Emmendingen, Germany) was employed.

Postoperative pain management

From postoperative day 1, patients received 1600 mg of acetaminophen four times daily as the baseline analgesic regimen. For breakthrough pain, 1000 mg of intravenous acetaminophen was administered. If the anticipated daily dose exceeded 4000 mg, intravenous pentazocine hydrochloride (15 mg) was administered instead.

Outcome measures and statistical analysis

The clinical experience of the operating surgeons (measured in years) was compared between groups. Postoperative hemorrhage was defined as bleeding requiring hemostatic intervention under general anesthesia. The incidence of hemorrhage was compared between groups. Additional outcomes included operative time, intraoperative blood loss, the frequency of rescue analgesia use, and the presence or absence of postoperative taste disturbances, which were assessed based on patient self-report during follow-up visits. 

Statistical analysis was performed using the Mann-Whitney U test for continuous variables and Fisher’s exact test for categorical variables, with a significance threshold of p < 0.05. All analyses were conducted using R software (The R Foundation for Statistical Computing, Vienna, Austria). Test statistics (e.g., U or χ² values) and p-values were reported for each comparison when applicable. To complement the interpretation of statistical significance, effect sizes were also calculated: for continuous variables, r values were derived from the Mann-Whitney U test; for categorical variables, Cramér’s V was computed from contingency tables. As Fisher’s exact test does not produce a test statistic, only the p-values are reported for those comparisons.

## Results

Patient characteristics

There were 85 patients in the Conventional Group and 52 in the Standardized Group (Preemptive Hemostasis Group). There were no significant differences between the groups in age, sex, or underlying diseases (p > 0.05). However, the incidence of previous peritonsillar abscess was significantly higher in the Standardized Group. Patient demographics are summarized in Table 2.

**Table 2:** Patient characteristics Note: Fisher’s exact test does not produce a test statistic; therefore, only p-values and effect sizes are presented. *Data are given as n (%), except for age, which is given as mean±SD

Variables		Conventional Group (n=85), n (%)*	Standardized Group (n=52), n (%)*	Test Used	Test Statistic	Effect Size	p value
Gender	Male	46 (54.1%)	21 (40.4%)	Fisher’s exact test	—	0.133	>0.05
	Female	39 (45.9%)	31 (59.6%)				
Age (years), mean±SD		32.8±13.9	40.0±16.3	Mann–Whitney U test	1656.5	0.194	0.0182
Indication for Tonsillectomy	Chronic tonsillitis	77 (90.6%)	33 (63.4%)	Fisher’s exact test	—	0.366	0.0001
	Chronic tonsillitis with previous peritonsillar abscess	1 (1.2%)	9 (17.3%)				
	IgA nephropathy	7 (8.2%)	9 (17.3%)				
	Inverted papilloma	0 (0%)	1 (1.9%)				

Surgical experience and perioperative outcomes

The surgical experience of the operating surgeons and perioperative outcomes are summarized in Table 3. The mean years of surgical experience were significantly greater in the Conventional group than in the Standardized Group (4.9 ± 2.4 years vs. 1.69 ± 1.69 years, p < 0.001). The mean operative time was significantly shorter in the Conventional Group compared to the Standardized Group (56.5 ± 23.8 minutes vs. 83.5 ± 33.3 minutes, p < 0.001). Intraoperative blood loss was significantly lower in the Standardized Group (0.42 ± 1.01 mL) than in the Conventional Group (8.31 ± 26.7 mL) (p = 0.0031). The length of hospital stay was also shorter in the Standardized Group (8.86 ± 1.24 days) than in the Conventional Group (10.1 ± 1.15 days) (p < 0.001). Postoperative bleeding occurred in eight patients in the Conventional Group, whereas no cases were observed in the Standardized Group (p = 0.0236). The mean number of rescue analgesic uses was significantly lower in the Standardized Group (0.67 ± 1.52) compared to the Conventional Group (1.23 ± 2.42) (p = 0.0178). There were no reports of persistent postoperative taste disturbances in either group.

**Table 3:** Surgical experience and perioperative outcome Note: Fisher’s exact test does not produce a test statistic; therefore, only p-values and effect sizes are presented. *Data given as mean±SD, except for Postoperative Bleeding, which is given as n (%)

Variable	Conventional Group (n=85), mean±SD*	Standardized Group (n=52), mean±SD*	Test Used	Test Statistic	Effect Size	p value
Years of Surgical Experience	4.9±2.4	1.69±1.69	Mann–Whitney U test	4137	0.73	0.001＞
Operative time	56.5±23.8	83.5±33.3		1017	0.444	0.001＞
Intraoperative blood loss	8.31±26.7	0.42±1.01		2757	0.207	0.0031
Length of hospital stay	10.1±1.15	8.86±1.24		3404	0.502	0.001＞
Postoperative bleeding, n (%)	8 (9.4%)	0 (0%)	Fisher’s exact test	—	0.195	0.0236
Number of rescue analgesic uses	1.23±2.42	0.67±1.52	Mann–Whitney U test	2625	0.168	0.0178

## Discussion

Postoperative hemorrhage remains one of the most serious complications following tonsillectomy and can be life-threatening [8]. Despite the introduction of novel hemostatic devices, the risk of bleeding and the need for reoperation under general anesthesia have not been completely eliminated [15]. Currently, no definitive preventive strategy has been established. Although intracapsular tonsillectomy, a less invasive technique, has gained attention, it does not entirely eliminate postoperative complications [16]. Furthermore, residual tonsillar tissue is sometimes undesirable in specific clinical settings. Therefore, there is an increasing need to establish a safe and reliable extracapsular tonsillectomy technique that minimizes complications. 

This study evaluated the efficacy of a surgical approach combining preemptive hemostasis, reduced energy output, and endoscopic assistance. Compared to the conventional technique, this method demonstrated superiority in several key outcomes, suggesting its potential to enhance surgical safety and educational value.

Effectiveness of device output limitation

Reduction in Postoperative Hemorrhage and Pain

In the Standardized Group, the incidence of postoperative hemorrhage requiring reoperation was 0%, which is markedly lower than previously reported rates of 3.8-7.9% [17,18]. Additionally, the frequency of rescue analgesic use was significantly reduced, indicating improved postoperative pain control. These outcomes are likely attributable to meticulous preemptive hemostasis and the use of low-output energy devices, which minimized excessive thermal injury.

Management of Thermal Injury

Effective vascular hemostasis typically occurs at temperatures around 60°C, where heat and pressure induce collagen denaturation to achieve coagulation [19]. Choi et al. have emphasized that maintaining tissue temperatures below 80°C is critical to minimizing collateral damage while ensuring hemostasis [20]. In contrast, excessive thermal exposure (>100°C) leads to tissue carbonization and protein denaturation, which may elevate the risk of postoperative bleeding [21]. In the present study, surgical energy output was initially limited to 12 W and was further reduced if macroscopic carbonization or vaporization was observed. This protocol aimed to avoid overt thermal injury and promote biologically favorable wound healing. Although previous studies, such as that by Baek et al., have demonstrated that a 15 W setting may offer clinical benefits over higher outputs in terms of reducing postoperative pain and bleeding [22], the optimal energy setting in tonsillectomy remains undetermined and likely depends on multiple real-time intraoperative factors. Actual tissue impedance varies depending on device type, electrode and cable condition, tissue thickness, and surgical field cleanliness [23], necessitating dynamic adjustment of energy output during surgery. Based on our clinical experience, even 15 W may be excessive in cases with low tissue resistance. Therefore, we standardized the initial bipolar energy output to ≤12 W as a conservative yet effective starting point to minimize thermal damage while maintaining adequate hemostasis. Further clinical studies are warranted to refine and individualize the optimal energy settings in bipolar-assisted tonsillectomy.

Significance of preemptive hemostasis and sharp dissection

In surgical techniques without preemptive hemostasis, ruptured vessels often retract into the tonsillar fossa, requiring extensive surface coagulation for bleeding control [24]. This can exacerbate thermal damage, worsen postoperative pain, and delay healing. In contrast, preemptive hemostasis improves visualization, enabling localized vascular control and allowing for sharp dissection along an accurate, fine plane. Compared to blunt dissection, sharp dissection is associated with reduced collateral tissue damage and more efficient wound healing [25-27]. Secondary postoperative hemorrhage typically occurs between postoperative days 5 and 10, coinciding with eschar detachment and exposure of incompletely healed vessels [28]. Therefore, minimizing excessive intraoperative carbonization is directly linked to the prevention of secondary bleeding. The present findings support the notion that combining preemptive hemostasis with appropriate coagulation and sharp dissection effectively reduces postoperative complications.

Benefits of endoscopic support and surgical standardization

Endoscopic supervision by senior surgeons played a crucial role in enhancing both patient safety and surgical education. The use of endoscopy allowed for real-time guidance without interfering with the operative autonomy of junior surgeons. Moreover, the ability to record procedures provided valuable educational material for postoperative review and training. Importantly, this approach utilizes equipment commonly available for endoscopic sinus surgery, making it both simple and widely applicable in various clinical settings. In contrast to microscope-assisted techniques, such as the microsurgical bipolar tonsillectomy described by Andrea in 1993, which enables excellent visualization and facilitates early identification of small vessels for precise hemostasis and minimal thermal injury [14], but inherently requires an operating microscope, our method was designed to be completed under direct vision by the primary surgeon. The endoscope was used solely as a supervisory and educational tool during training, allowing senior surgeons to provide real-time guidance while promoting independent skill development. This design aims to enable surgeons, after mastering the procedure under endoscopic supervision during training, to later perform safe and consistent tonsillectomies without the need for endoscopic or other specialized support. It is particularly advantageous in settings where access to dedicated equipment is limited or where surgeons are expected to operate independently. By combining direct visualization with low-output energy techniques and structured supervision, our standardized protocol fosters safe, reproducible, and self-reliant surgical practices. In this respect, endoscopic support offers practical and educational advantages over microscope-dependent techniques, especially in training environments focused on skill acquisition and procedural consistency.

Reduction of Postoperative Complications, Hospital Stay, and Healthcare Resource Utilization

The combination of low energy output and endoscopic assistance resulted in reduced postoperative hemorrhage, decreased pain, and shorter hospital stays. These improvements not only promote faster patient recovery but also enhance healthcare system efficiency. Notably, these favorable outcomes were achieved without reliance on costly, single-use hemostatic devices, highlighting the potential for cost-effective surgical practice.

Operative Time and Educational Context

The longer operative time observed in the Standardized Group may be attributed in part to the fact that surgeries were performed by relatively inexperienced surgeons who required intraoperative instruction and supervision. While this naturally extended the duration of the procedures, it reflects the educational intent of the protocol and contributes to the safe acquisition of standardized, reproducible surgical skills.

Study limitations and future perspectives

This study has several limitations. First, in the Conventional Group, detailed settings of the energy devices were determined by the surgeon's discretion and were not systematically recorded. This lack of standardization may have led to variability in thermal injury and hemostatic performance, potentially affecting postoperative outcomes such as bleeding and pain. As a result, it introduces a limitation in the internal validity of the intergroup comparison. Second, because the intervention combined three components, output limitation, preemptive hemostasis, and endoscopic assistance, it remains unclear which factor had the greatest impact. Although the initial energy setting was empirically set at ≤12 W, further research is needed to determine the optimal output. Future multicenter collaborative studies will be essential to validate the present findings and to establish a standardized protocol aimed at reducing postoperative complications in tonsillectomy.

## Conclusions

The technique incorporating preemptive hemostasis and low-power energy devices under endoscopic assistance appears to be effective in reducing postoperative hemorrhage requiring general anesthesia, as well as minimizing intraoperative blood loss and postoperative pain. Additionally, this approach may enhance the educational experience of junior surgeons and suggests the potential for improving procedural safety. However, given the observational and non-randomized design of this study, the findings should be interpreted with caution. Further multicenter prospective studies are needed to validate and generalize these results. Nevertheless, this study provides valuable insights for the refinement of standard techniques in palatine tonsillectomy.
